# The Symptomatology of Stridor in Diseases of Children

**Published:** 1914-08

**Authors:** 


**The Symptomatology of Stridor in Diseases of Children.** Bach, in Zeitschrift für Kinderheilkunde, April 4, 1914, found the Rőntgen ray examination of great value in cases caused by endothoracal processes such as hyperplastic thymus, tuberculosis of the bronchial lymphnodes, abscesses, infantile asthmatic bronchitis, and deviations of the trachea.—C. G. L-W.

